# Prevalence of High-Burden Medical Conditions Among Young and Middle-Aged Adults With Pediatric-Onset Medical Conditions: Findings From US Private and Public Administrative Claims Data

**DOI:** 10.15171/ijhpm.2019.62

**Published:** 2019-07-29

**Authors:** Daniel G. Whitney

**Affiliations:** Department of Physical Medicine and Rehabilitation, University of Michigan, Ann Arbor, MI, USA.

**Keywords:** Clinical Epidemiology, Pediatric-Onset Medical Conditions, Noncommunicable Diseases, United States

## Abstract

Adults with pediatric-onset medical conditions (POMCs) are susceptible to early development of high-burden medical conditions. However, research pertaining to this topic is lacking, which is vital information that could assist in health benefit planning and administration. The purpose of this study was to determine the prevalence of high-burden medical conditions among privately and publicly insured adults with POMCs, as compared to adults without POMCs, from the US. Data from 2016 were extracted from Optum Clinformatics® Data Mart (private insurance) and a random 20% sample from Medicare fee-for-service (public insurance). International Classification of Diseases, Tenth Revision, Clinical Modification (ICD-10-CM) diagnosis codes were used to identify 18–64-year-old beneficiaries with POMCs, as well as several high-burden medical conditions, including pain, fracture, mood affective disorders, anxiety disorders, ischemic heart diseases, cerebrovascular diseases, hypertensive and other cardiovascular diseases, type 2 diabetes, osteoporosis, osteoarthritis, chronic obstructive pulmonary diseases, liver diseases, and cancer. Privately and publicly insured adults with POMCs had higher prevalence of all medical conditions compared to adults without POMCs. Publicly insured adults with POMCs had higher prevalence of all medical conditions compared to privately insured adults with POMCs, except for the lower prevalence of pain and cancer. When stratified by the category of POMCs (eg, musculoskeletal, circulatory), privately and publicly insured groups tended to have higher prevalence of most (private) or all (public) medical conditions compared to adults without POMCs. Adults with POMCs have higher prevalence of several high-burden medical conditions compared to adults without POMCs. This health disparity was present regardless of insurance coverage, but was generally more pronounced for public vs. private insured adults with POMCs.

## Background

Noncommunicable diseases are growing public health issues,^1^ and represent a considerable source for early mortality^2^ and economic burden.^3^ The burden of noncommunicable diseases may disproportionally affect populations with disabilities, especially if the disability originated in childhood.

Pediatric-onset medical conditions (POMCs) refer to a group of congenital or acquired conditions that originate at any point from conception through adolescence, and consist of impairments in behavioral, emotional, mental, intellectual, or physical function, or are associated with altered development or abnormal physiological processes. Very little clinical and research attention has been given to individuals with POMCs as they become adults, despite the anticipated growth of the adult population with POMCs.^4^ Importantly, individuals with POMCs have complicated healthcare needs throughout the lifespan. This increases risk for early development of high-burden medical conditions, which refer to conditions that lead to high financial cost, excess morbidity, early mortality, or poor quality of life, and include pain, fracture, and noncommunicable diseases.^5-8^ Therefore, a comprehensive understanding of the clinical epidemiology of high-burden medical conditions among young and middle-aged adults with POMCs is needed. Such information could inform healthcare policy-makers and clinical practice guidelines that promote early and cost-effective care for the aging population with POMCs. This study sought to inform these efforts by providing recent US national level estimates of the prevalence of several high-burden medical conditions for adults with POMCs that have private or public health insurance, as compared to adults without POMCs. We hypothesized that adults with POMCs would have higher prevalence of all high-burden medical conditions compared to adults without POMCs. We also hypothesized that this disparity would be greater for publicly insured adults with POMCs than privately insured adults with POMCs.

## Methods

### Data Sources

Data for this study were extracted from private and public administrative claims data from the year 2016. Optum Clinformatics^®^ Data Mart Database (OptumInsight^TM^, Eden Prairie, MN, USA) provided de-identified information for privately insured beneficiaries (commercial or Medicare Advantage plans). A random 20% sample of the Medicare fee-for-service database provided de-identified information for publicly insured beneficiaries.

### Sample Selection

Beneficiaries that were 18 to 64 years of age, had 12 full months of continuous enrollment, and had at least one medical service utilization in 2016 were included. Medicare beneficiaries were excluded if they were covered by Health Maintenance Organization plans because of incomplete claims. Medical diagnoses of all POMCs and medical conditions were identified using the International Classification of Diseases, Tenth Revision, Clinical Modification (ICD-10-CM) codes, and are presented in Table S1 (see Supplementary file 1).

A single claim in any position (medical file in Optum; inpatient, outpatient, and carrier file in Medicare) for POMCs was used to identify these beneficiaries. The single claim-based definition has shown good accuracy for identifying pediatric-onset conditions using administrative claims data, with sensitivity of 99% and positive predictive value of 79%.^9^ It is not possible to determine if some POMCs developed in childhood or adulthood (eg, spinal cord injury) using a single year of claims data. Therefore, only conditions with known childhood origins were included. These conditions were then grouped into the following POMC subcategories: musculoskeletal system; neurodevelopmental; circulatory system; nervous system; genital organs; other chromosomal abnormalities; urinary system; respiratory and digestive systems; and malformations of the eye, ear, face, and neck. Beneficiaries of private insurance without any medical claims for POMCs represented the group without POMCs. The group without POMCs were ascertained through Optum to enhance representativeness of the sample of adults without POMCs, because criteria for enrollment in Medicare under 65 years of age requires permanent disability.

### Medical Conditions

Prevalent medical conditions were ascertained using the same procedure to identify POMCs. Medical conditions were selected with guidance from the literature on disease, disability, and mortality,^1,2,5,10-12^ and included pain, all-cause fracture, and several noncommunicable diseases: mood affective disorders; anxiety disorders; ischemic heart diseases; cerebrovascular diseases; hypertensive and other cardiovascular diseases; type 2 diabetes mellitus; osteoporosis; osteoarthritis; chronic obstructive pulmonary diseases; chronic kidney diseases; liver diseases; and malignant cancer anywhere in the body.

### Participant Characteristics

Data on age and gender were extracted as they were reported in the same manner from Optum and Medicare. Other confounding variables were not considered for covariate adjustment to limit bias for reasons such as they had not been reported in both data sources (eg, education level), they had not been reported in the same manner (eg, race), or they had missing data on over 20% of the group (eg, income).

### Statistical Analysis

All measures were summarized using SAS version 9.4 (SAS Institute, Inc., Cary, NC). Data were presented for adults without POMCs and with POMCs stratified by insurance coverage (private, public). Then, data were presented for adults with POMCs after stratifying by each POMC category for private and public insured beneficiaries. 95% CIs were calculated for the POMC groups. A sensitivity analysis was performed to determine prevalence estimates of medical conditions, but with the requirement of having at least 2 claims from separate days for each POMC and medical condition category.

## Results

Participant characteristics for adults without POMCs (n = 5 415 475) and adults with POMCs that had private (n = 121 446) or public (n = 119 654) insurance is presented in Table 1. There were differences in the prevalence of POMC categories between private and public insurance. Notably, the publicly insured group had considerably (>3 times) higher prevalence of neurodevelopmental POMCs and considerably (>3 times) lower prevalence of POMCs of the genital organs compared to the privately insured group.

**Table 1:** Characteristics and Prevalence of High-Burden Medical Conditions Among Adults With and Without POMCs

	**Without POMCs**	**With POMCs**	**With POMCs**
	**Private Insurance** **(n = 5 415 475)**	**Private Insurance** **(n = 121 446)**	**Public Insurance** **(n = 119 654)**
**Descriptive characteristics**	Point estimate	Point estimate	Point estimate
Age, mean (SD)	43.8 (12.9)	44.3 (13.6)	46.9 (11.9)
18-40 years, %	40.3	44.1	30.9
41-64 years, %	59.7	55.9	69.1
Gender, %			
Female	53.8	55.6	46.8
Male	46.2	44.4	53.2
**POMC categories, %**			
Musculoskeletal system		27.6	10.8
Neurodevelopmental		14.1	56.7
Circulatory system		20.1	9.4
Nervous system		9.1	18.6
Genital organs		5.1	0.9
Other chromosomal abnormalities		4.5	9.6
Urinary system		10.3	5.9
Respiratory and digestive system		10.0	4.0
Malformation of eye, ear, face, or neck		4.8	2.1

Abbreviations: POMCs, pediatric-onset medical conditions; SD, standard deviation. Note: Individuals may have more than one POMC and can be represented across multiple POMC categories.

Prevalence of high-burden medical conditions among adults with and without POMCs is presented in Table 2. Privately and publicly insured adults with POMCs had higher prevalence of all medical conditions compared to adults without POMCs. Publicly insured adults with POMCs had higher prevalence of all medical conditions compared to privately insured adults with POMCs, except for the lower prevalence of pain and cancer.

**Table 2:** Prevalence of High-Burden Medical Conditions Among Adults With and Without POMCs

	**Without POMCs**	**With POMCs**	**With POMCs**
	**Private Insurance** **(n = 5415475)**	**Private Insurance** **(n = 121446)**	**Public Insurance** **(n = 119654)**
	**%**	**% (95% CI)**	**% (95% CI)**
Pain	30.1	45.2 (44.9, 45.5)	42.6 (42.3, 42.9)
Fracture	2.6	5.2 (5.1, 5.3)	7.8 (7.6, 8.0)
Mood affective disorders	11.0	20.8 (20.6, 21.0)	41.2 (40.9, 41.5)
Anxiety disorders	15.1	25.5 (25.3, 25.7)	37.9 (37.6, 38.2)
Ischemic heart diseases	3.5	8.7 (8.5, 8.9)	11.4 (11.2, 11.6)
Cerebrovascular diseases	1.5	6.3 (6.2, 6.4)	8.6 (8.4, 8.8)
Hypertensive and other cardiovascular diseases	25.4	37.8 (37.5, 38.1)	50.6 (50.3, 50.9)
Type 2 diabetes	10.0	14.5 (14.3, 14.7)	24.9 (24.7, 25.1)
Osteoporosis	1.3	3.4 (3.3, 3.5)	7.4 (7.3, 7.5)
Osteoarthritis	7.7	15.8 (15.6, 16.0)	19.3 (19.1, 19.5)
Chronic obstructive pulmonary disease	0.7	1.8 (1.7, 1.9)	12.0 (11.8, 12.2)
Chronic kidney disease	1.8	6.6 (6.5, 6.7)	11.4 (11.2, 11.6)
Liver diseases	2.9	7.3 (7.2, 7.4)	8.2 (8.0, 8.4)
Cancer	3.8	6.6 (6.5, 6.7)	5.9 (5.8, 6.0)

Abbreviation: POMCs, pediatric-onset medical conditions.

Prevalence of high-burden medical conditions for each of the 9 POMC categories is presented in Table 3. Compared to adults without POMCs, most categories of POMCs from the privately insured group (7 to 9 categories) tended to have higher prevalence of all medical conditions. Compared to adults without POMCs, most categories of POMCs from the publicly insured group (8 to 9 categories) tended to have higher prevalence of all medical conditions. Finally, compared to the privately insured group of adults with POMCs, the publicly insured group had higher prevalence of all high-burden medical conditions for each POMC category, except for the similar or lower prevalence between neurodevelopmental POMCs for cerebrovascular diseases, POMCs of the nervous system for pain, ischemic heart diseases, cerebrovascular diseases, liver diseases, and cancer, and POMCs of other chromosomal abnormalities for pain, liver diseases, and cancer.

**Table 3:** High-Burden Medical Conditions Among Adults (18-64 Years) Stratified by the Category of POMCs

	**Musculoskeletal System**	**Neurodevelopmental**	**Circulatory System**	**Nervous System**	**Genital Organs**	**Chromosomal Abnormalities**	**Urinary System**	**Respiratory Digestive Systems**	**Eye, Ear, Face, or Neck**
Sample size, n									
Private insurance	33 566	17 149	24 393	11 021	6164	5518	12 477	12 190	5811
Public insurance	12 910	67 791	11 276	22 276	1070	11 431	6998	4744	2454
Pain, % (95% CI)									
Private insurance	63.4 (62.9, 63.9)	27.8 (27.1, 28.5)	39.8 (39.2, 40.4)	46.0 (45.1, 46.9)	31.9 (30.7, 33.1)	27.2 (26.0, 28.4)	47.3 (46.4, 48.2)	45.3 (44.4, 46.2)	34.4 (33.2, 35.6)
Public insurance	74.1 (73.3, 74.9)	31.8 (31.4, 32.2)	55.2 (54.3, 56.1)	39.1 (38.5, 39.7)	56.7 (53.7, 59.7)	25.7 (24.9, 26.5)	62.8 (61.7, 63.9)	66.6 (65.3, 67.9)	45.1 (43.1, 47.1)
Fracture, % (95% CI)									
Private insurance	7.7 (7.4, 8.0)	4.8 (4.5, 5.1)	4.1 (3.9, 4.3)	5.7 (5.3, 6.1)	2.3 (1.9, 2.7)	3.6 (3.1, 4.1)	4.5 (4.1, 4.9)	4.7 (4.3, 5.1)	3.5 (3.0, 4.0)
Public insurance	12.2 (11.6, 12.8)	7.3 (7.1, 7.5)	8.6 (8.1, 9.1)	7.5 (7.2, 7.8)	7.9 (6.3, 9.5)	5.2 (4.8, 5.6)	9.0 (8.3, 9.7)	10.1 (9.2, 11.0)	7.5 (6.5, 8.5)
Mood affective disorders, % (95% CI)							
Private insurance	19.2 (18.8, 19.6)	35.4 (34.7, 36.1)	18.3 (17.8, 18.8)	24.7 (23.9, 25.5)	13.5 (12.6, 14.4)	13.7 (12.8, 14.6)	18.8 (18.1, 19.5)	20.6 (19.9, 21.3)	14.5 (13.6, 15.4)
Public insurance	44.2 (43.3, 45.1)	45.1 (44.7, 45.5)	39.7 (38.8, 40.6)	30.8 (30.2, 31.4)	45.4 (42.4, 48.4)	22.1 (21.3, 22.9)	39.8 (38.7, 40.9)	48.7 (47.3, 50.1)	34.4 (32.5, 36.3)
Anxiety disorders, % (95% CI)							
Private insurance	24.2 (23.7, 24.7)	37.0 (36.3, 37.7)	24.1 (23.6, 24.6)	27.1 (26.3, 27.9)	20.1 (19.1, 21.1)	18.7 (17.7, 19.7)	23.6 (22.9, 24.3)	26.6 (25.8, 27.4)	19.0 (18.0, 20.0)
Public insurance	42.8 (41.9, 43.7)	39.9 (39.5, 40.3)	39.8 (38.9, 40.7)	29.0 (28.4, 29.6)	44.2 (41.2, 47.2)	23.0 (22.2, 23.8)	39.8 (38.7, 40.9)	49.4 (48.0, 50.8)	31.8 (30.0, 33.6)
Ischemic heart diseases, % (95% CI)								
Private insurance	5.4 (5.2, 5.6)	4.7 (4.4, 5.0)	19.9 (19.4, 20.4)	5.6 (5.2, 6.0)	2.9 (2.5, 3.3)	3.2 (2.7, 3.7)	10.9 (10.4, 11.4)	8.4 (7.9, 8.9)	4.4 (3.9, 4.9)
Public insurance	14.7 (14.1, 15.3)	6.8 (6.6, 7.0)	33.8 (32.9, 34.7)	5.8 (5.5, 6.1)	14.5 (12.4, 16.6)	4.4 (4.0, 4.8)	26.9 (25.9, 27.9)	21.7 (20.5, 22.9)	11.1 (9.9, 12.3)
Cerebrovascular diseases, % (95% CI)							
Private insurance	3.1 (2.9, 3.3)	6.2 (5.8, 6.6)	15.0 (14.6, 15.4)	7.9 (7.4, 8.4)	1.7 (1.4, 2.0)	2.8 (2.4, 3.2)	5.5 (5.1, 5.9)	4.0 (3.7, 4.3)	2.9 (2.5, 3.3)
Public insurance	9.0 (8.5, 9.5)	6.2 (6.0, 6.4)	24.7 (23.9, 25.5)	7.7 (7.3, 8.1)	8.6 (6.9, 10.3)	4.3 (3.9, 4.7)	13.7 (12.9, 14.5)	11.9 (11.0, 12.8)	6.1 (5.2, 7.0)
Hypertensive and other cardiovascular diseases, % (95% CI)							
Private insurance	32.2 (31.7, 32.7)	32.3 (31.6, 33.0)	49.0 (48.4, 49.6)	35.8 (34.9, 36.7)	19.5 (18.5, 20.5)	21.1 (20.0, 22.2)	57.7 (56.8, 58.6)	40.1 (39.2, 41.0)	28.8 (27.6, 30.0)
Public insurance	59.3 (58.5, 60.1)	45.4 (45.0, 45.8)	71.4 (70.6, 72.2)	42.4 (41.8, 43.0)	58.6 (55.6, 61.6)	28.8 (28.0, 29.6)	83.4 (82.5, 84.3)	67.2 (65.9, 68.5)	50.3 (48.3, 52.3)
Type 2 diabetes, % (95% CI)									
Private insurance	12.9 (12.5, 13.3)	16.7 (16.1, 17.3)	15.7 (15.2, 16.2)	12.7 (12.1, 13.3)	7.7 (7.0, 8.4)	11.7 (10.9, 12.5)	18.9 (18.2, 19.6)	15.1 (14.5, 15.7)	14.3 (13.4, 15.2)
Public insurance	28.9 (28.1, 29.7)	23.0 (22.7, 23.3)	33.5 (32.6, 34.4)	15.5 (15.0, 16.0)	32.2 (29.4, 35.0)	17.2 (16.5, 17.9)	37.1 (36, 38.2)	33.3 (32.0, 34.6)	28.9 (27.1, 30.7)
Osteoporosis, % (95% CI)									
Private insurance	4.0 (3.8, 4.2)	3.1 (2.8, 3.4)	2.9 (2.7, 3.1)	4.3 (3.9, 4.7)	1.0 (0.8, 1.2)	3.6 (3.1, 4.1)	3.6 (3.3, 3.9)	4.4 (4.0, 4.8)	2.2 (1.8, 2.6)
Public insurance	11.0 (10.5, 11.5)	7.3 (7.1, 7.5)	7.4 (6.9, 7.9)	10.9 (10.5, 11.3)	6.7 (5.2, 8.2)	7.5 (7.0, 8.0)	8.0 (7.4, 8.6)	10.5 (9.6, 11.4)	7.1 (6.1, 8.1)
Osteoarthritis, % (95% CI)									
Private insurance	25.1 (24.6, 25.6)	9.4 (9.0, 9.8)	13.6 (13.2, 14.0)	14.1 (13.5, 14.7)	6.0 (5.4, 6.6)	7.6 (6.9, 8.3)	15.9 (15.3, 16.5)	15.9 (15.3, 16.5)	9.8 (9.0, 10.6)
Public insurance	40.0 (39.2, 40.8)	13.7 (13.4, 14.0)	26.0 (25.2, 26.8)	15.5 (15.0, 16.0)	26.5 (23.9, 29.1)	12.2 (11.6, 12.8)	29.2 (28.1, 30.3)	32.5 (31.2, 33.8)	20.3 (18.7, 21.9)
Chronic obstructive pulmonary disease, % (95% CI)							
Private insurance	1.4 (1.3, 1.5)	1.2 (1.0, 1.4)	2.6 (2.4, 2.8)	1.6 (1.4, 1.8)	0.7 (0.5, 0.9)	0.9 (0.7, 1.1)	2.4 (2.1, 2.7)	2.7 (2.4, 3.0)	1.1 (0.8, 1.4)
Public insurance	18.0 (17.3, 18.7)	8.6 (8.4, 8.8)	24.0 (23.2, 24.8)	7.5 (7.2, 7.8)	15.1 (13.0, 17.2)	4.5 (4.1, 4.9)	21.1 (20.1, 22.1)	27.1 (25.8, 28.4)	11.5 (10.2, 12.8)
Chronic kidney disease, % (95% CI)							
Private insurance	3.2 (3.0, 3.4)	5.1 (4.8, 5.4)	6.4 (6.1, 6.7)	4.3 (3.9, 4.7)	2.3 (1.9, 2.7)	4.0 (3.5, 4.5)	27.7 (26.9, 28.5)	5.3 (4.9, 5.7)	3.3 (2.8, 3.8)
Public insurance	11.6 (11.0, 12.2)	6.7 (6.5, 6.9)	21.5 (20.7, 22.3)	5.7 (5.4, 6.0)	15.5 (13.3, 17.7)	6.8 (6.3, 7.3)	57.2 (56.0, 58.4)	18.2 (17.1, 19.3)	11.3 (10.0, 12.6)
Liver diseases, % (95% CI)							
Private insurance	4.9 (4.7, 5.1)	4.3 (4.0, 4.6)	7.5 (7.2, 7.8)	4.6 (4.2, 5.0)	4.0 (3.5, 4.5)	4.6 (4.0, 5.2)	17.2 (16.5, 17.9)	15.4 (14.8, 16.0)	4.3 (3.8, 4.8)
Public insurance	10.1 (9.6, 10.6)	5.8 (5.6, 6.0)	13.5 (12.9, 14.1)	5.2 (4.9, 5.5)	12.7 (10.7, 14.7)	5.0 (4.6, 5.4)	22.1 (21.1, 23.1)	22.9 (21.7, 24.1)	7.1 (6.1, 8.1)
Cancer, % (95% CI)									
Private insurance	5.2 (5.0, 5.4)	3.5 (3.2, 3.8)	7.1 (6.8, 7.4)	5.4 (5.0, 5.8)	4.6 (4.1, 5.1)	5.7 (5.1, 6.3)	11.9 (11.3, 12.5)	10.5 (10.0, 11.0)	6.6 (6.0, 7.2)
Public insurance	7.7 (7.2, 8.2)	4.0 (3.9, 4.1)	9.1 (8.6, 9.6)	4.4 (4.1, 4.7)	10.7 (8.8, 12.6)	3.4 (3.1, 3.7)	14.1 (13.3, 14.9)	13.2 (12.2, 14.2)	7.3 (6.3, 8.3)

Abbreviation: POMCs, pediatric-onset medical conditions. Note: Individuals may have more than one POMC and can be represented across multiple POMC categories.

The results of the sensitivity analysis requiring at least two claims from different days for each category of POMCs and medical conditions are presented in Table S2 for adults with and without POMCs and Table S3 after stratifying by the type of POMC category (see Supplementary file 1). Interpretations were unchanged for adults with and without POMCs. Interpretations were largely unchanged for most categories of POMCs from the privately and publicly insured group compared to controls.

## Discussion

The chief finding here is that young and middle-aged adults with POMCs had higher prevalence of several high-burden medical conditions compared to adults without POMCs. While this was evident for both privately and publicly insured adults with POMCs, these health disparities were more pronounced for the publicly insured group with POMCs. These are critical findings, because these high-burden medical conditions are major contributors to the global and national burden of disease and mortality,^2,13-15^ and are typically associated with advanced aging. Further, many of these high-burden medical conditions can be prevented, delayed, or better managed to reduce patient and caregiver burden. Taken together, these findings highlight the need for earlier clinical, rehabilitative, and healthcare policy efforts to improve awareness and access to quality healthcare, such as screening strategies, for these populations to allow for earlier detection of these high-burden medical conditions.

In the current study, we also found that adults with POMCs of a particular biological system may be at greater risk of high-burden medical conditions of that same biological system. Notably, POMCs of the musculoskeletal system had the highest prevalence of fracture, osteoporosis, and osteoarthritis; POMCs of the circulatory system had the highest prevalence of ischemic heart diseases, cerebrovascular diseases, and hypertensive and other cardiovascular diseases; and POMCs of the urinary system had the highest prevalence of chronic kidney diseases. This information could assist clinical care for adults with POMCs, by allowing clinicians to target preventative and rehabilitation care specific to the needs of their patients with POMCs. This information could also assist in health benefit planning, by allowing administrators to allocate healthcare resources based on the POMC demographics of health plans.

A major strength of the current study is the large sample of adults with POMCs from both private and public insurance databases, thus enhancing external validity.^16^ Gathering data on clinical populations can be challenging, and little is known about the healthful aging process for individuals with POMCs across the lifespan. Another major strength of this study is the comprehensive assessment of several high-burden medical conditions, with many representing major contributors to the global and national burden of disease and mortality.^2,13-15^ The present findings are robust and could improve clinical care and inform new research examining health disparities and healthcare delivery for individuals with POMCs.

The limitations must be discussed. First, while Optum and Medicare databases are nationwide, Optum coverage is more predominant in Southern and Western regions of the United States, which may bias estimates because of regional differences in chronic disease prevalence.^17^ Second, the primary analysis used a single claim to identify beneficiaries with POMCs and medical conditions. While validation studies have shown that at least 2 claims for a medical condition improves ability to identify beneficiaries with that medical condition,^9,18^ single claim-based algorithms have been reported to have moderate-to-high positive predictive value (~80%) to detect POMCs,^9^ or specificity up to 96% to detect a variety of medical conditions;^19,20^ however, the accuracy of identifying conditions using claims data depends on the length of the study period^19^ and the medical condition examined.^9,19-21^ In light of this, a sensitivity analysis was performed that used at least two claims from different days to identify all POMCs and medical conditions. The results revealed the same conclusions as the primary analysis. When taken together, given the short study period of 12 months, the selected methodology to identify medical conditions using a single claim is likely sufficient to detect that the current data is evidence of health disparities among adults with POMCs, as compared to adults without POMCs. Third, the present study excluded individuals that did not have service utilization in 2016, which may have biased results. However, these excluded individuals that had insurance coverage may be somewhat healthy since they did not require a medical encounter in 2016, thus potentially biasing results in the present study to be more conservative estimates. Fourth, it is not possible using claims-based data from a single year to determine if certain conditions developed in childhood or later in life (eg, spinal cord injury, cancer, depression). Fifth, data were from a single year and longer study periods and longitudinal research designs could provide more robust findings and implications for research, practice, and policy. Lastly, the present study presented descriptive statistics rather than inferential statistics, and therefore did not account for potential confounding factors, such as indicators of socioeconomic status.

## Conclusion

Adults with POMCs have higher prevalence of several high-burden medical conditions compared to adults without POMCs. This health disparity was present for both private and public insurance coverage, but was generally more pronounced for public vs. private insured adults with POMCs. Study findings are critical, because many of these high-burden medical conditions are associated with advanced aging, and may be prevented, delayed, or better managed with adequate clinical knowledge. These findings highlight the need for clinical and public health strategies to implement earlier detection and preventive medical services for noncommunicable diseases and other high-burden medical conditions for adult populations with POMCs. Future research is needed to examine how these high-burden medical condition profiles associate with mortality and quality of life for adults with POMCs.

## Ethical issues

Since data are de-identified, consent to participate was not required and the University IRB approved this study as non-regulated.

## Competing interests

Author declares that he has no competing interests.

## Author’s contribution

DGW is the single author of the paper.

## Supplementary files


Supplementary file 1 contains Tables S1-S3.
