# Supplementary material for: Prevalence of High-Burden Medical Conditions Among Young and Middle-Aged Adults With Pediatric-Onset Medical Conditions: Findings From US Private and Public Administrative Claims Data
Source: Int J Health Policy Manag. 2019 Jul 29;8(11):629–35. doi: 10.15171/ijhpm.2019.62 (PMC6885858; doi:10.15171/ijhpm.2019.62)
Supplement: Supplementary file 1 — contains Tables S1-S3. [file ijhpm-8-629-s001.pdf]

Supplementary file 1

Table S1. International Classification of Diseases, Tenth Revision, Clinical Modification (ICD-10) codes used to identify all diagnoses.

Pediatric-onset disability categories and specific conditions	ICD-10 codes
**Musculoskeletal system**	
Congenital deformities of hip or feet; congenital musculoskeletal deformities of head, face, spine and chest; other congenital musculoskeletal deformities; polydactyly; syndactyly; reduction defects of upper, lower, or unspecified limb; other congenital malformations of limb(s); other congenital malformations of skull and face bones; congenital malformations of spine and bony thorax; osteochondrodysplasia with defects of growth of tubular bones and spine; other osteochondrodysplasias; congenital malformations of musculoskeletal system, not elsewhere classified	Q65-79 families
Juvenile arthritis	M08 family
Other disorders of bone	
Physeal arrest	M89.1 family
Other disorders of bone development and growth	M89.2 family
**Neurodevelopmental**	
Intellectual disabilities, mild, moderate, severe, profound, other, and unspecified	F70-73, F78, F79
Specific developmental disorders of speech and language, scholastic skills, motor function; pervasive developmental disorders; other and unspecified disorders of psychological development	F80 family, F81 family, F82, F84 family, F88, F99
**Circulatory system**	
Congenital malformations of the cardiac chambers, cardiac septa, pulmonary and tricuspid valves, aortic and mitral valves; other congenital malformations of the heart; congenital malformations of great arteries, great veins; other congenital malformations of peripheral vascular system or circulatory system	Q20-28 families

**Nervous system**	
Encephalocele; microcephaly; congenital hydrocephalus; spina bifida; other congenital malformations of brain, spinal cord, or nervous system	Q01 family, Q02, Q03-7 families
Cerebral palsy	G80 family
Juvenile epilepsy	G40.B family
**Genital organs**	
Congenital malformations of ovaries, fallopian tubes, broad ligaments, uterus, and cervix; undescended and ectopic testicle; hypospadias; other congenital malformations of female or male genital organs; indeterminate sex and pseudohermaphroditism	Q50-56 families
**Other chromosomal abnormalities, not classified elsewhere**	
Down syndrome; Trisomy 18 and Trisomy 13; other trisomies and partial trisomies of the autosomies, not elsewhere classified; monosomies and deletions from the autosomes, not elsewhere classified; balanced rearrangements and structural markers, not elsewhere classified; Turner's syndrome; other sex chromosome abnormalities, female or male phenotype, not elsewhere classified; other chromosome abnormalities	Q90-93 families, Q95-99 families
**Urinary system**	
Renal agenesis and other reduction defects of kidney; cystic kidney disease; congenital obstructive defects of renal pelvis and congenital malformations of ureter; other congenital malformations of kidney	Q60-63 families
**Respiratory and digestive systems**	
Congenital malformations of nose, larynx, trachea or bronchus, lung; other congenital malformations of respiratory system; cleft lip and/or palate; other congenital malformations of tongue, mouth, or pharynx; congenital malformations of esophagus; other congenital malformations of upper alimentary tract; congenital absence, atresia and stenosis of small or large intestine; other congenital malformations	Q30-45 families

of intestine; congenital malformations of gallbladder, bile ducts, or liver; other congenital malformations of digestive system	
**Malformations of the eye, ear, face, and neck**	
Congenital malformations of eyelid, lacrimal apparatus, or orbit; anophthalmos, microphthalmos, or macrophthalmos; congenital lens malformations; congenital malformations of anterior or posterior segment of eye; other congenital malformations of eye; congenital malformations of ear causing impairment of hearing; other congenital malformations of ear, face, or neck	Q10-18 families
**<u>High-burden medical conditions</u>**	
**Pain**	
Central pain syndrome; other chronic pain; chronic pain syndrome; dorsalgia, including panniculitis affecting regions of the neck and back, radiculopathy, cervicalgia, sciatica, lumbago with sciatica, low back pain, pain in thoracic spine, other or unspecified dorsalgia; pain in joint, including shoulder, elbow, wrist, hand, hip, knee, ankle/foot	G890, G8929, G894, M54 family, M255 family
**Fracture**	
Osteoporosis with current pathological fracture; fracture of skull and facial bones, cervical vertebra and other parts of neck, rib(s), sternum, and thoracic spine, lumbar spine and pelvis, shoulder and upper arm, forearm, wrist and hand, femur, lower leg, including ankle, foot and toe, except ankle	M80 family, S02 family, S12 family, S22 family, S32 family, S42 family, S52 family, S62 family, S72 family, S82 family, S92 family
**Mood affective disorders**	
Manic episode; bipolar disorder; major depressive disorder, single episode or recurrent; persistent mood [affective] disorders; unspecified mood [affective] disorders	F30-34 families, F39
**Anxiety disorders**	
Phobic anxiety disorders; other anxiety disorders; obsessive-compulsive disorder; reaction to severe stress, and adjust disorders;	F40-45 families, F48 family

dissociative and conversion disorders; somatoform disorders; other nonpsychotic mental disorders	
**Ischemic heart diseases**	
Angina pectoris; acute myocardial infarction; subsequent ST elevation (STEMI) and non-ST elevation (NSTEMI) myocardial infarction; other acute ischemic heart diseases; chronic ischemic heart disease	I20-22 families, I24 family, I25 family
**Cerebrovascular diseases**	
Nontraumatic subarachnoid or intracerebral hemorrhage; other and unspecified nontraumatic intracranial hemorrhage; cerebral infarction; occlusion and stenosis of precerebral or cerebral arteries, not resulting in cerebral infarction; other cerebrovascular diseases; cerebrovascular disorders in diseases classified elsewhere; sequelae of cerebrovascular disease	I60-63 families, I65-69 families
**Hypertensive and other cardiovascular diseases**	
Essential (primary) hypertension; hypertensive heart, chronic kidney disease, or heart and chronic kidney disease; secondary hypertension; hypertensive crisis; heart failure; peripheral atherosclerosis	I10-13 families, I15 family, I16 family, I50 family, I70 family
**Type 2 diabetes mellitus**	E11 family
**Osteoporosis**	
With or without current pathological fracture	M80 family, M81 family
**Osteoarthritis (OA)**	
Poly OA; hip OA; knee OA; OA of the first carpometacarpal joint; other and unspecified OA	M15-19 families
**Chronic obstructive pulmonary diseases**	
Simple and mucopurulent chronic bronchitis; unspecified chronic bronchitis; emphysema; other chronic obstructive pulmonary disease	J41-44 families
**Chronic kidney diseases**	
Stage I-V; end stage renal disease; chronic kidney disease, unspecified	N18 family
**Liver diseases**	

Alcoholic liver disease; toxic liver disease; hepatic failure, not elsewhere classified; chronic hepatitis, not elsewhere classified; fibrosis and cirrhosis of liver; other inflammatory liver diseases; other diseases of liver; liver disorders in diseases classified elsewhere	K70-76 families, K77
**Malignant cancer**	
Malignant neoplasms of lip, oral cavity, or pharynx, digestive organs, respiratory and intrathoracic organs, bone and articular cartilage, mesothelial and soft tissue, breast, female or male genital organs, urinary tract, eye, brain, or other parts of central nervous system, thyroid or other endocrine glands, ill-defined, other secondary, or unspecified sites, neuroendocrine tumors, lymphoid, hematopoietic, or related tissue; melanoma and other malignant neoplasms of skin	C00-26 families, C30-41 families, C43-58 families, C60-80 families, C7A family, C7B family, C81-96 families

Table S2. Prevalence of high-burden medical conditions among adults with and without pediatric-onset medical conditions (POMCs). Results of sensitivity analysis using 2+ claims to identify POMCs and medical conditions.

	Without POMCs	With POMCs	With POMCs
	Private insurance (n=5,482,235)	Private insurance (n=54,686)	Public insurance (n=69,434)
	%	% (95% CI)	% (95% CI)
Pain	21.2	35.5 (35.1, 35.9)	27.7 (27.4, 28.0)
Fracture	1.8	3.9 (3.7, 4.1)	5.4 (5.2, 5.5)
Mood affective disorders	7.1	17.8 (17.5, 18.1)	31.2 (30.8, 31.5)
Anxiety disorders	9.3	20.2 (19.8, 20.5)	26.1 (25.8, 26.5)
Ischemic heart diseases	2.5	6.6 (6.3, 6.8)	5.8 (5.6, 6.0)
Cerebrovascular diseases	0.8	4.8 (4.6, 5.0)	4.5 (4.4, 4.7)
Hypertensive and other CVD	18.3	32.6 (32.2, 33.0)	39.4 (39.1, 39.8)
Type 2 diabetes	8.1	12.2 (11.9, 12.5)	18.4 (18.1, 18.6)
Osteoporosis	0.6	2.2 (2.0, 2.3)	5.0 (4.8, 5.2)
Osteoarthritis	4.4	10.2 (10.0, 10.5)	9.7 (9.5, 9.9)

Chronic obstructive pulmonary disease	1.7	4.4 (4.3, 4.6)	6.1 (5.9, 6.3)
Chronic kidney disease	1.3	6.4 (6.2, 6.6)	8.3 (8.1, 8.5)
Liver diseases	1.2	3.4 (3.3, 3.6)	3.3 (3.2, 3.4)
Cancer	2.9	4.4 (4.2, 4.6)	3.7 (3.6, 3.9)

CI, confidence interval; CVD, cardiovascular diseases.

Table S3. Prevalence of high-burden medical conditions among adults (18-64 years) stratified by the category of pediatric-onset medical conditions (POMCs). Results of sensitivity analysis using 2+ claims to identify POMCs and medical conditions.

	Musculoskeletal system	Neurodevelopmental	Circulatory system	Nervous system	Genital organs	Chromosomal abnormalities	Urinary system	Respiratory digestive systems	Eye, ear, face, or neck
Sample size, n									
Private insurance	14,231	10,384	12,181	6,378	2,181	2,569	5,497	2,583	1,294
Public insurance	5,370	40,332	5,195	14,918	350	7,587	3,184	1,349	751
	% (95% CI)	% (95% CI)	% (95% CI)	% (95% CI)	% (95% CI)	% (95% CI)	% (95% CI)	% (95% CI)	% (95% CI)
Pain									
Private insurance	56.6 (55.8, 57.4)	18.6 (17.9, 19.3)	29.1 (28.3, 29.9)	37.6 (36.4, 38.8)	21.8 (20.1, 23.5)	18.5 (17.0, 20.0)	35.6 (34.3, 36.9)	37.4 (35.5, 39.3)	26.4 (24.0, 28.8)
Public insurance	66.3 (65.0, 67.6)	17.7 (17.3, 18.1)	41.2 (39.9, 42.5)	29.2 (28.5, 29.9)	42.0 (36.8, 47.2)	17.7 (16.8, 18.6)	47.9 (46.2, 49.6)	54.3 (51.6, 57.0)	33.6 (30.2, 37.0)
Fracture									
Private insurance	5.6 (5.2, 6.0)	4.0 (3.6, 4.4)	2.6 (2.3, 2.9)	4.8 (4.3, 5.3)	1.8 (1.2, 2.4)	3.4 (2.7, 4.1)	3.3 (2.8, 3.8)	3.5 (2.8, 4.2)	2.2 (1.4, 3.0)

Public insurance	8.3 (7.6, 9.0)	5.3 (5.1, 5.5)	5.6 (5.0, 6.2)	5.3 (4.9, 5.7)	6.3 (3.8, 8.8)	4.4 (3.9, 4.9)	5.5 (4.7, 6.3)	7.0 (5.6, 8.4)	5.1 (3.5, 6.7)
Mood affective disorders									
Private insurance	14.6 (14.0, 15.2)	33.2 (32.3, 34.1)	13.5 (12.9, 14.1)	19.8 (18.8, 20.8)	9.0 (7.8, 10.2)	12.1 (10.8, 13.4)	13.6 (12.7, 14.5)	17.1 (15.6, 18.6)	9.5 (7.9, 11.1)
Public insurance	33.3 (32.0, 34.6)	35.5 (35.0, 36.0)	27.8 (26.6, 29.0)	24.3 (23.6, 25.0)	29.7 (24.9, 34.5)	18.0 (17.1, 18.9)	28.5 (26.9, 30.1)	38.2 (35.6, 40.8)	25.0 (21.9, 28.1)
Anxiety disorders									
Private insurance	17.4 (16.8, 18.0)	34.1 (33.2, 35.0)	17.1 (16.4, 17.8)	19.7 (18.7, 20.7)	13.7 (12.3, 15.1)	13.9 (12.6, 15.2)	15.6 (14.6, 16.6)	22.0 (20.4, 23.6)	12.8 (11.0, 14.6)
Public insurance	30.8 (29.6, 32.0)	28.8 (28.4, 29.2)	26.4 (25.2, 27.6)	20.0 (19.4, 20.6)	24.9 (20.4, 29.4)	15.9 (15.1, 16.7)	27.3 (25.8, 28.8)	36.5 (33.9, 39.1)	20.6 (17.7, 23.5)
Ischemic heart diseases									
Private insurance	3.4 (3.1, 3.7)	3.6 (3.2, 4.0)	15.0 (14.4, 15.6)	3.7 (3.2, 4.2)	2.1 (1.5, 2.7)	2.5 (1.9, 3.1)	8.5 (7.8, 9.2)	6.4 (5.5, 7.3)	3.3 (2.3, 4.3)
Public insurance	8.6 (7.9, 9.3)	3.0 (2.8, 3.2)	23.0 (21.9, 24.1)	3.1 (2.8, 3.4)	9.1 (6.1, 12.1)	2.5 (2.1, 2.9)	19.2 (17.8, 20.6)	13.5 (11.7, 15.3)	6.9 (5.1, 8.7)
Cerebrovascular diseases									
Private insurance	1.8 (1.6, 2.0)	4.0 (3.6, 4.4)	12.1 (11.5, 12.7)	4.4 (3.9, 4.9)	1.0 (0.6, 1.4)	2.3 (1.7, 2.9)	3.4 (2.9, 3.9)	3.1 (2.4, 3.8)	2.4 (1.6, 3.2)
Public insurance	4.8 (4.2, 5.4)	2.9 (2.7, 3.1)	17.7 (16.7, 18.7)	4.0 (3.7, 4.3)	5.4 (3.0, 7.8)	2.6 (2.2, 3.0)	8.5 (7.5, 9.5)	6.9 (5.5, 8.3)	3.9 (2.5, 5.3)
Hypertensive and other CVD									
Private insurance	24.9 (24.2, 25.6)	26.9 (26.0, 27.8)	41.9 (41.0, 42.8)	31.3 (30.2, 32.4)	13.8 (12.4, 15.2)	19.5 (18.0, 21.0)	56.3 (55.0, 57.6)	36.0 (34.1, 37.9)	24.1 (21.8, 26.4)

Public insurance	47.2 (45.9, 48.5)	35.3 (34.8, 35.8)	60.3 (59.0, 61.6)	34.9 (34.1, 35.7)	51.7 (46.5, 56.9)	22.5 (21.6, 23.4)	79.7 (78.3, 81.1)	57.2 (54.6, 59.8)	43.9 (40.4, 47.4)
Type 2 diabetes									
Private insurance	10.0 (9.5, 10.5)	15.0 (14.3, 15.7)	12.5 (11.9, 13.1)	10.5 (9.7, 11.3)	5.9 (4.9, 6.9)	11.8 (10.6, 13.0)	15.4 (14.4, 16.4)	12.7 (11.4, 14.0)	12.4 (10.6, 14.2)
Public insurance	21.3 (20.2, 22.4)	17.7 (17.3, 18.1)	25.1 (23.9, 26.3)	11.4 (10.9, 11.9)	23.7 (19.2, 28.2)	14.4 (13.6, 15.2)	28.5 (26.9, 30.1)	28.5 (26.1, 30.9)	26.6 (23.4, 29.8)
Osteoporosis									
Private insurance	2.7 (2.4, 3.0)	2.1 (1.8, 2.4)	1.6 (1.4, 1.8)	3.0 (2.6, 3.4)	0.6 (0.3, 0.9)	3.2 (2.5, 3.9)	2.2 (1.8, 2.6)	2.9 (2.3, 3.5)	1.6 (0.9, 2.3)
Public insurance	7.0 (6.3, 7.7)	5.2 (5.0, 5.4)	4.4 (3.8, 5.0)	7.5 (7.1, 7.9)	4.6 (2.4, 6.8)	5.3 (4.8, 5.8)	4.4 (3.7, 5.1)	5.9 (4.6, 7.2)	2.7 (1.5, 3.9)
Osteoarthritis									
Private insurance	18.5 (17.9, 19.1)	5.8 (5.4, 6.2)	7.3 (6.8, 7.8)	9.6 (8.9, 10.3)	3.2 (2.5, 3.9)	5.7 (4.8, 6.6)	9.9 (9.1, 10.7)	9.0 (7.9, 10.1)	5.8 (4.5, 7.1)
Public insurance	28.5 (27.3, 29.7)	6.3 (6.1, 6.5)	13.8 (12.9, 14.7)	8.9 (8.4, 9.4)	13.7 (10.1, 17.3)	7.2 (6.6, 7.8)	16.1 (14.8, 17.4)	18.1 (16.0, 20.2)	11.6 (9.3, 13.9)
Chronic obstructive pulmonary disease									
Private insurance	2.9 (2.6, 3.2)	4.1 (3.7, 4.5)	6.4 (6.0, 6.8)	4.8 (4.3, 5.3)	1.5 (1.0, 2.0)	2.8 (2.2, 3.4)	4.8 (4.2, 5.4)	8.3 (7.2, 9.4)	2.6 (1.7, 3.5)
Public insurance	11.5 (10.6, 12.4)	3.9 (3.7, 4.1)	15.4 (14.4, 16.4)	4.6 (4.3, 4.9)	10.3 (7.1, 13.5)	2.9 (2.5, 3.3)	14.4 (13.2, 15.6)	19.4 (17.3, 21.5)	8.1 (6.1, 10.1)
Chronic kidney disease									
Private insurance	2.2 (2.0, 2.4)	4.2 (3.8, 4.6)	5.1 (4.7, 5.5)	3.5 (3.0, 4.0)	1.4 (0.9, 1.9)	4.5 (3.7, 5.3)	32.2 (31, 33.4)	6.4 (5.5, 7.3)	2.9 (2.0, 3.8)

Public insurance	8.0 (7.3, 8.7)	4.6 (4.4, 4.8)	15.6 (14.6, 16.6)	4.1 (3.8, 4.4)	12.9 (9.4, 16.4)	5.4 (4.9, 5.9)	63.0 (61.3, 64.7)	17.2 (15.2, 19.2)	9.7 (7.6, 11.8)
Liver diseases									
Private insurance	2.1 (1.9, 2.3)	2.2 (1.9, 2.5)	4.0 (3.7, 4.3)	2.3 (1.9, 2.7)	2.3 (1.7, 2.9)	2.6 (2.0, 3.2)	7.5 (6.8, 8.2)	11.6 (10.4, 12.8)	1.7 (1.0, 2.4)
Public insurance	4.7 (4.1, 5.3)	2.3 (2.2, 2.4)	6.5 (5.8, 7.2)	2.1 (1.9, 2.3)	6.3 (3.8, 8.8)	2.6 (2.2, 3.0)	11.7 (10.6, 12.8)	12.8 (11.0, 14.6)	3.5 (2.2, 4.8)
Cancer									
Private insurance	3.5 (3.2, 3.8)	2.5 (2.2, 2.8)	4.7 (4.3, 5.1)	3.4 (3.0, 3.8)	3.9 (3.1, 4.7)	4.1 (3.3, 4.9)	8.9 (8.1, 9.7)	7.6 (6.6, 8.6)	5.8 (4.5, 7.1)
Public insurance	5.4 (4.8, 6.0)	2.8 (2.6, 3.0)	5.6 (5.0, 6.2)	3.0 (2.7, 3.3)	8.0 (5.2, 10.8)	2.0 (1.7, 2.3)	10.3 (9.2, 11.4)	10.3 (8.7, 11.9)	7.1 (5.3, 8.9)

CI, confidence interval. Individuals may have more than one POMC and can be represented across multiple POMC categories.
